# Effect of supplemented paprika oleoresin solution on the physicochemical properties and shelf-life of boiled pork sausages with nitrite reduction

**DOI:** 10.5713/ab.24.0258

**Published:** 2024-08-23

**Authors:** Geon Ho Kim, Koo Bok Chin

**Affiliations:** 1 Department of Animal Science, Chonnam National University, Gwangju 61186, Korea

**Keywords:** Physicochemical Properties, Paprika Oleoresin, Pork Boiled Sausage, Shelf-Life, Sodium Nitrite

## Abstract

**Objective:**

This study was performed to evaluate the quality characteristics of reduced-nitrite boiled pork sausages (BPSs) with paprika oleoresin solution (POS) to compensate for the reduced sodium nitrite (NaNO_2_).

**Methods:**

POS was prepared by diluting paprika oleoresin with sunflower seed oil at a ratio of 1:20. BPSs were subjected to four different treatments: reference (REF), BPS added with 150 ppm NaNO_2_; control (CTL), BPS added with 37.5 ppm NaNO_2_; treatment 1 (TRT1), BPS added with 37.5 ppm NaNO_2_ and 0.1% POS; treatment 2 (TRT2), BPS added with 75 ppm NaNO_2_ and 0.1% POS).

**Results:**

The pH values of CTL were lower than those of other treatments. The a* values of TRT1 were higher than those of CTL, and those of REF were lower than those of TRT1 and TRT2. The b* values of TRT1 and TRT2 were higher than those of REF and CTL. The total plate counts of CTL were the highest among all treatments, and *Enterobacteriaceae* counts of CTL and TRT1 on the 14th day were higher than those of REF and TRT2.

**Conclusion:**

The combination of 75 ppm NaNO_2_ and 0.1% POS to BPS during storage had an antimicrobial effect similar to that of adding 150 ppm NaNO_2_. Thus, POS can be used to reduce the use of NaNO_2_ in meat products.

## INTRODUCTION

Sodium nitrite (NaNO_2_) is a curing agent used in meat processing to impart a specific pink color to meat through the formation of nitrosohemochrome. Sodium nitrite also inhibits the growth of microorganisms such as *Clostridium botulinum* in meat products [1]. Furthermore, it imparts a specific flavor to meat products and inhibits lipid oxidation and the production of off-odors [2]. Therefore, sodium nitrite is among the most important ingredients in meat products.

However, nitrite has a high chance of forming the carcinogen N-nitrosamine [3]. Currently, most consumers are interested in healthy foods. Although consumers have a negative perception of meat products containing high amounts of nitrite, several studies reported that consumers had positive purchase intention toward new meat products with natural compounds and reduced-nitrite level [4,5].

Consequently, recognizing the consumer’s demands for healthy foods, the meat industry is focusing on studies to find an alternative to nitrite for improving the color and storage of meat products.

Paprika (*Capsicum annuum* var. *angulosum*) has a high contents of an antioxidants, such as ascorbic acid, tocopherol, and phenolic compounds. It can prevent cancer and coronary artery disease due to its naturally high antioxidant activity [6]. Carotenoids, which are red- and yellow-colored pigments present in paprika, are typical antioxidants widely used in the food industry. Capsanthin, a carotenoid, has been reported to increase the redness of meat products [7]. Paprika oleoresin is a color-producing ingredient that is obtained by processing paprika fruit and used as an additive for sauces, soups, and meat-based meals in food industries [8]. It has high color stability owing to its high carotenoid content, and therefore, it is enough to impart the coloring effect of paprika [9]. The addition of paprika oleoresin to meat products can help reduce or replace the content of sodium nitrite (NaNO_2_). However, studies in this area are limited. Therefore, this study was performed to evaluate the quality and storage characteristics of reduced-nitrite boiled pork sausages (BPSs) supplemented with paprika oleoresin solution (POS).

## MATERIALS AND METHODS

### Materials

Raw pork ham and back fat used were obtained from 1st grade castrated three-way crossbreed (Landrace×Yorkshire× Duroc) pigs. They were purchased from a retail meat market (Hyundai Retail Meat Market, Gwangju, Korea). After removing connective tissues and external fat, raw hams were minced using a meat chopper (M-12S; Hankook Fujee Industries Co., Ltd., Hwaseong, Korea), vacuum packaged, and stored frozen until use. Back fat was cut off from the surface of the raw material and ground with a meat chopper, and stored at −20°C. Paprika oleoresin was obtained from Kalsec (Kalamazoo, MI, USA). The sunflower seed oil used to make POS (pH 9.81±0.07) was obtained from a local supermarket, and paprika oleoresin and sunflower seed oil were diluted in a 1:20 ratio.

### Preparation of pork sausages

The formulation used for preparing the BPS is presented in Table 1. Three batches of BPSs (total approximately 2,000 g, consisting of 1,200 g ground pork ham and 400 g pork back fat for each treatment) with different curing ingredients including NaNO_2_ or POS concentrations in the present study. BPSs were manufactured following to modified method of Lee and Chin [10]. After the raw meat was mixed with curing ingredients in ice water using a bowl cutter (K-15; Talsa, Valencia, Spain), the fat and POS were added and the mixture was emulsified. Raw sausage batter was stuffed into polyvinylidene chloride and cooked in a water bath (WB-22; Daihan Scientific Co., Ltd., Seoul, Korea) until the internal temperature of the sausages reached 71.8°C. The cooked sausages were cooled and stored at 10°C until analysis.

### pH determination

pH was measured five times for each treatment group using a solid pH meter (Model 120; Mettler-Toledo, Greifeense, Switzerland) and the results were averaged. Calibration of pH values was performed to adjust the standard curve based on the pH 4.01 and pH 7.00 buffer solutions.

### Color measurement

The color values of the PSs were determined by cutting the samples to a thickness of 1.5 cm and measuring the lightness (CIE L*), redness (CIE a*), yellowness (CIE b*) of the treated samples sis times using a Minolta Color Reader (CR-10; Minolta Co., Ltd, Osaka, Japan). The results were averaged. The CIE color value of the standard white plate was L* = 94.8, a* = 1.0, b* = 0.1.

### Purge loss

Purge loss (PL, %) was measured to determine the weight lost by packaged samples during refrigerated storage. The PSs were taken out from the package and the amount of exudate moisture in the samples was measured. PL was calculated using the following formula:


$$\text{PL }(\%)=\frac{Packaged\;sample\;weight\;(g)-Unpackaged\;sample\;weight\;(g)}{Packaged\;sample\;weight\;(g)}\times 100$$

### Cooking loss

Cooking loss (CL, %) was calculated by substituting the measured weight of the sausage before and after heating into the following formula:


$$\text{CL }(\%)=\frac{Raw\;sample\;weight\;(g)-Heated\;sample\;weight\;(g)}{Raw\;sample\;weight\;(g)}\times 100$$

### Expressible moisture

Samples for expressible moisture (EM, %) were prepared by cutting the sausage into a rectangular parallelepiped shape of 1.5 g. The sample was wrapped in three pieces of quadrupled Whatman #3 filter paper, placed in a conical tube, and centrifuged at 1,660×g for 15 min (VS-5500; Vision Science Co., Ltd., Daejeon, Korea). Subsequently, the amount of moisture expressed from the sample in the filter paper was measured and substituted into the following formula:


$$\text{EM }(\%)=\frac{Weight\;of\;moisture\;expreessed\;(g)}{Sample\;weight\;(g)}\times 100$$

### Texture profile analysis

Ten sausage samples of 1.25 cm in diameter and 1.3 cm in height were prepared for texture profile analysis (TPA). The hardness (gf), springiness (mm), gumminess, chewiness, and cohesiveness of each sample were measured using an Instron Universal Machine (Model 3344; Instron, Canton, MA, USA) with compression probe at speed of 300 mm/min and load cell of 500 N for a two-bite test, and the mean values were obtained.

### Microbial counts

Microbial counts were performed by mixing 10 g of homogenized sausage samples with 90 mL of sterile double-distilled water and diluting them to the appropriate proportion. Total plate count (TPC) and the violet red bile (VRB) agar plates were used for the determining the total number of viable bacteria and *Enterobacteriaceae*, respectively. After the inoculation of the mixture, the medium was incubated at 37°C in an incubator for 48 h and the results were expressed as a log CFU/g.

### Residual nitrite

Residual nitrite was determined using the AOAC method [11], with slight modifications. Approximately 5 g of ground sausage samples were mixed with 300 mL of double distilled (dd)-water and heated for 1 h at 100°C in a constant temperature water bath (WB-22; Daihan Scientific Co., Ltd., Korea). After filtration, the samples were diluted using dd-water to obtain a final volume of 500 mL. A 2.5 mL of sulfanilamide was added to 25 mL of this mixture, vortexed, and allowed to react for 5 min. Then, 2.5 mL of N-(1-Naphthyl) ethylene dihydrochloride was added to 25 mL of this mixture, vortexed, and allowed to react for 5 min. The absorbance was measured at a wavelength of 540 nm using a spectrophotometer (UV-1601; Shimadzu, Kyoto, Japan). The measured absorbance was assigned to a standard curve obtained by measuring the absorbance of the nitrite solution to determine the amount of residual nitrite in the samples.

### Thiobarbituric acid reactive substances

The thiobarbituric acid reactive substances (TBARS) value was measured using the method described by Sinnhuber and Yu [12]. Ground sausage sample (2 g), 3 mL of thiobarbituric acid, and 17 mL of trichloroacetic acid (100 mg/mL) were vortexed, homogenized, and heated for 30 min at 100°C in a water bath (WB-22; Daihan Scientific Co., Ltd., Korea). Subsequently, the samples were cooled at room temperature and then 5 mL of the supernatant of the sample and 5 mL of chloroform were vortexed together for 1 min and then centrifuged at 1,660×g (VS-5500; Vision Science Co., Ltd., Korea) for 5 min. Subsequently, 3 mL of the supernatant of the sample and 3 mL of petroleum ether were vortexed together for 1 min and then centrifuged at 1,660×g for 10 min. The absorbance of the reaction product was measured at a wavelength of 532 nm using a spectrophotometer. The TBARS values were derived based on standard curve using tetraethoxypropane solution.

### Statistical analysis

All analyses were performed in triplicate for each batch (n = 3). The mean and standard deviation of the results were calculated using IBM SPSS Statistics 23 (SPSS Inc., Chicago, IL, USA). The results of CL, EM, and TPA were subjected to one-way analysis of variance (ANOVA) to investigate the differences between treatments. Statistical comparisons of other experiments except for those parameters were conducted using two-way ANOVA (treatments×storage time). Post-hoc analysis was performed using Duncan’s multiple range test at a significance level of 0.05.

## RESULTS AND DISCUSSION

### pH and color values

**Figure 1:**
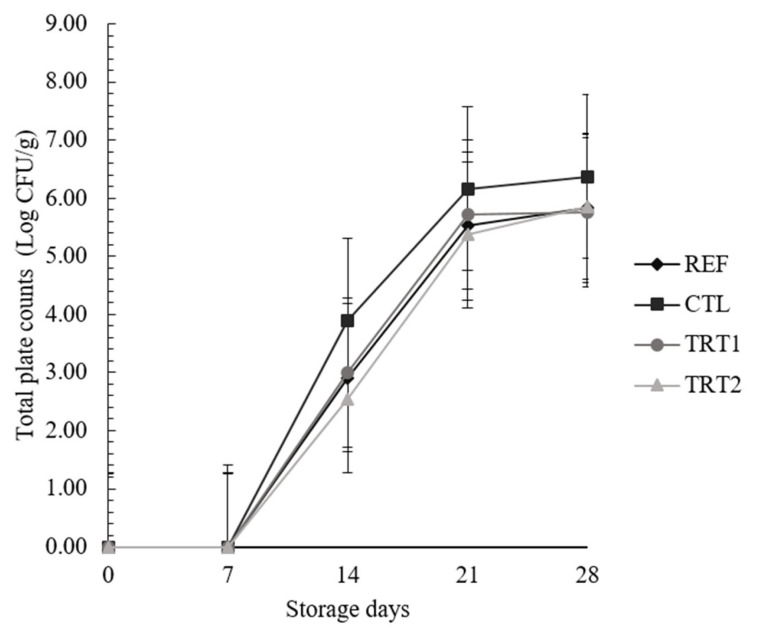
Changes in the total plate counts of boiled pork sausages with different levels of sodium nitrite and paprika oleoresin solution. Each values represents the mean±standard deviation, n = 3. Treatments: REF, boiled pork sausage (BPS) with 150 ppm sodium nitrite (NaNO_2_); CTL, BPS with 37.5 ppm NaNO_2_; TRT1, PS with 37.5 ppm NaNO_2_ and 0.1% paprika oleoresin solution (POS, 5% paprika oleoresin+95% sunflower seed oil); TRT2, PS with 75 ppm NaNO_2_ and 0.1% POS.

As shown in Table 2, control (CTL) had the highest pH values among all treatments (p<0.05), and those of reference (REF), treatment 1 (TRT1), and treatment 2 (TRT2) were not different (p>0.05). The pH values of all treatments including CTL tended to decrease with increasing storage time (p<0.05). This might be related to microbial growth in the meat products during refrigerated storage. The TPC of CTL were higher than those of all other treatments in this study (Figure 1). Langlois and Kemp [13] reported that the pH values of the vacuum-packaged sliced ham decreased due to the lactic acid and various organic acids produced by the increasing number of *Lactobacillus* during storage, which is similar to the results observed in the present study.

Table 2 shows the color values of the PSs. The CIE L* values of CTL were the highest among all treatments (p<0.05), followed by those of REF, TRT1, and TRT2, which were not different (p>0.05). These results suggested that the CIE L* values of PSs were affected by the different sodium nitrite and POS levels. In a study by Froehlich et al [14], the L* values of low-nitrite ham were higher than those of treatments with higher levels of nitrite (0, 50, 100, and 150 ppm), which was similar to the results of the present study. Although CTL and treatments with POS had the same sodium nitrite content, the CIE a* values of TRT1 were higher than those of CTL (p<0.05). Despite having lower nitrite levels, TRT1 and TRT2 showed higher CIE a* values than REF (p<0.05). However, CTL with 37.5 g/kg nitrite showed lower CIE a* values than REF owing to its lower nitrite content [15]. TRT1 and TRT2 showed higher CIE a* values than CTL and REF because they contained 0.1% POS in addition to 37.5 or 75 ppm of nitrite. The CIE b* values of TRT1 and TRT2 were not different (p>0.05) but were higher than those of REF and CTL (p<0.05). The b* values of REF and CTL, which did not contain POS, were not different (p>0.05) but those of REF were higher than those of CTL (p<0.05). This is because the addition of POS increased the CIE b* values. The increase in CIE a* and b* values in treatments with POS was reported in a previous model study [16], but it did not appear that those of the PSs with 37.5 and 75 ppm nitrite were not different, and this result was partially due to the addition of sugar and spices. Furthermore, paprika oleoresin resulted in the similar increases in CIE a* values of reduced-nitrite pork sausages as compared to the dried paprika powder [17]. These results suggested that the addition of POS increased redness (a*) values of color development in reduced-nitrite meat products.

### Microbial counts

**Figure 2:**
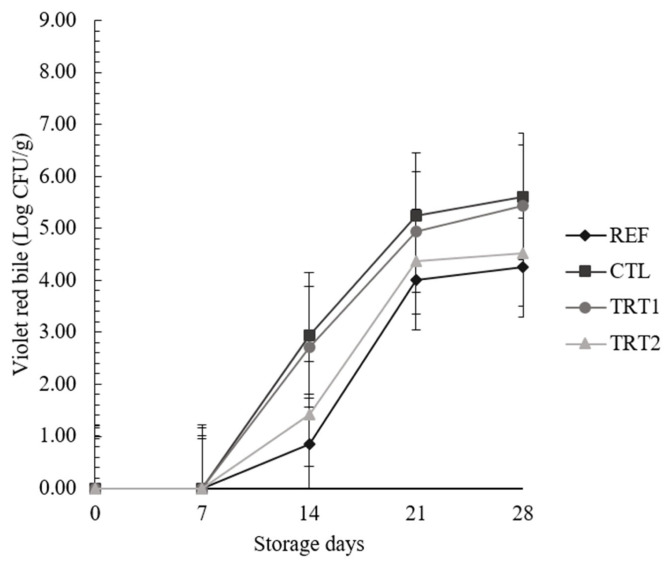
Changes in the *Enterobacteriaceae* counts of boiled pork sausages with different levels of sodium nitrite and paprika oleoresin solution. Each values represents the mean±SD, n = 3. Treatments: REF, boiled pork sausage (BPS) with 150 ppm sodium nitrite (NaNO_2_); CTL, BPS with 37.5 ppm NaNO_2_; TRT1, PS with 37.5 ppm NaNO_2_ and 0.1% paprika oleoresin solution (POS, 5% paprika oleoresin+95% sunflower seed oil); TRT2, PS with 75 ppm NaNO_2_ and 0.1% POS.

As shown in Figure 1, the TPC of CTL were the highest among all treatments (p<0.05), and those of all other treatments did not differ (p>0.05). This might be attributed to the antimicrobial effects of sodium nitrite, which resulted in CTL, which had lower sodium nitrite, having higher TPCs than REF. Nitric oxide formed from nitrite can inhibit the growth of gram-positive microorganisms [18]. However, TRT1 and TRT2, which had the same level of nitrite as CTL, showed TPCs similar to those of REF, which had, higher nitrite levels (p>0.05). These results suggested that the addition of POS could decrease the TPCs of PSs compared to that of the CTL. The *Enterobacteriaceae* counts in the PSs are shown in Figure 2. There was an interaction between the storage days and treatments (p<0.05). *Enterobacteriaceae* were not detected in any treatment and the CTL from days 0 to 7; however, they were detected in CTL and TRT1 after 14 days of storage, and their levels were higher than those in the REF and TRT2 (p<0.05). Although the *Enterobacteriaceae* counts of the treatments were not different on day 21 (p>0.05), the *Enterobacteriaceae* counts of CTL and TRT1 were higher than those of REF and TRT2 on day 28 (p<0.05), and those of REF and TRT2 were not different during the storage time (p>0.05). Nielsen [19] reported that *Brocothrix thermosphacta* and *Enterobacteriaceae* counts in cured pork loin with 200 ppm nitrite were less than those in treatments with 100 ppm nitrite owing to the antimicrobial effects of nitrite. Bang et al [20] reported that sausages with 154 ppm of nitrite in combination with salt showed higher retardation of *Enterobacteriaceae* than those with salt alone, and the combination of salt and nitrite could control the *Enterobacteriaceae* counts and prolong the storage time of meat products. Thus, POS could be antimicrobial agents for development of reduced-nitrite meat product.

### Purge loss

The PL of the BPSs is shown in Table 3. No differences were observed in PL among all treatments and storage days (p> 0.05). The addition of sodium nitrite and POS did not affect the water-holding capacity during storage, and consequently, the PL was also not affected. Chung et al [21] reported that the addition of kkuaripepper powder (*Capsicum annum* L.) increased the water-holding capacity of pork sausages, and the improvement in water-holding capacity might be affected by the increase in emulsification due to the addition of 1% to 5% kkuaripepper powder. In our study, the addition of oleoresin extracted from pepper, such as POS, did not affect the water-holding capacity of pork sausages unlike the pepper powder used in their study.

### Thiobarbituric acid reactive substances

As shown in Table 3, the TBARS values were not different among the treatments (p>0.05). It indicated that the addition of nitrite and POS did not affect lipid oxidation during refrigerated storage. According to Kim and Chin [16], model pork sausages without nitrite had higher TBARS values than those with regular (150 ppm) or reduced (37.5 or 75 ppm) levels of nitrite during refrigerated storage. Kim et al [22] reported that pork patties with pork patties with 0.323% paprika oleoresin had lower TBARS values than the control. The present study indicated that the addition of nitrite in combination with paprika oleoresin decreased the TBARS values, which is different from the results of the previous studies. This might be because the inhibition of lipid oxidation by nitrite or POS differed depending on the type of ingredients used in the meat products.

### Residual nitrite

Table 4 shows the residual nitrite contents of the boiled PSs supplemented with POS. Because the residual nitrite content was influenced by the interaction between treatments and storage time, all the data were separated as treatments at a particular storage time or storage times in a treatment (p<0.05). The residual nitrite content in all treatments decreased on day 3 and tended to plateau or continued to decrease thereafter depending on the treatments (p<0.05). The residual nitrite contents of REF were higher than those of the other treatments (p<0.05) from the initial storage until day 28, suggesting that the amount of nitrite (150 ppm) added had a significant effect on the residual nitrite at the end of storage. Jeon and Choi [23] reported that the residual nitrite contents in pork patties with 150 ppm of nitrite were higher than those of treatments with 50 and 100 ppm nitrite until 6 wks of storage. These results suggested that the residual nitrite levels during storage differed depending on the amount of nitrite initially added to the sausages, and reduced-nitrite BPSs added with POS might reduce potential risk compared to regular level (150 ppm).

### Cooking loss, expressible moisture, and texture profile analysis

Table 5 shows the results of CL (%) from the PSs. No differences in CL were observed among all treatments (p>0.05). In a previous study by Kim and Chin [16], CLs of reduced-level nitrite (37.5 to 75 ppm) model PSs supplemented with POS were not different from those of the samples with regular-level nitrite (150 ppm) which is similar to the results of the present study. These results indicated that changes in the POS or nitrite levels did not affect the CL of PSs.

There were no differences in the EM (%) of PSs among all treatments (Table 5) (p>0.05). Thus, the addition of POS did not affect the water-holding capacity of PSs. In a study by Bázan-Lugo et al [24], EMs of pork sausages added with 1.5% or 2% paprika powder were higher than those of the control. Because the addition of paprika powder increased the moisture content of the PSs, their EMs also increased. In contrast, in the present study, the EM values did not improve upon the addition of paprika because an oleoresin-type paprika pigment was added to the PSs.

As shown in Table 6, the TPA parameters such as hardness, springiness, gumminess, chewiness, and cohesiveness were not different among the treatments (p>0.05). Revilla and Quintana [25] reported that the additional level of paprika (1.8% to 2.8%) did not affect textural properties such as adhesiveness, chewiness, cohesiveness, and gumminess of Chorizo. These results suggested that the textural properties of meat products were not affected by the addition of paprika pigment because the addition levels of sodium nitrite and paprika oleoresin were too small to create a difference in the TPA parameters. Therefore, the application of POS might be used without negative effects on functional properties of pork sausages with reduction of nitrite.

## CONCLUSION

The addition of POS increased redness values of reduced-nitrite BPSs more than those with regular-level of NaNO_2_. During refrigerated storage, POS inhibited the microbial growth of BPS with the reduction of NaNO_2_. Since the actual additional and residual level of NaNO_2_ were decreased, reduced-nitrite BPSs with POS reduced potential health risk. Furthermore, POS had no negative effects of water-holding capacity and textural properties. In conclusion, the addition of POS could help the development of heathier-meat products with the reduction of NaNO_2_ by antimicrobial activity and color stability.

## Figures and Tables

**Table 1:** The formulation for manufacturing boiled pork sausages with different levels of sodium nitrite and paprika oleoresin solution

Items	Treatments^1)^			

	REF	CTL	TRT1	TRT2
Ingredients (%)				
Meat	60.0	60.0	60.0	60.0
Fat	20.0	20.0	20.0	20.0
Water	15.05	15.05	15.05	15.05
Non-meat ingredients				
Sodium chloride	1.50	1.50	1.50	1.50
Sodium tripolyphosphate	0.40	0.40	0.40	0.40
Sodium erythorbate	0.05	0.05	0.05	0.05
Sugar	1.00	1.00	1.00	1.00
Spice	1.00	1.00	1.00	1.00
Corn syrup	1.00	1.00	1.00	1.00
Paprika oleoresin solution	0.00	0.00	0.10	0.10
Total	100.01	100.00	100.1	100.11

1) Treatments: REF, boiled pork sausage (BPS) with 150 ppm sodium nitrite (NaNO_2_); CTL, BPS with 37.5 ppm NaNO_2_; TRT1, PS with 37.5 ppm NaNO_2_ and 0.1% paprika oleoresin solution (POS, 5% paprika oleoresin + 95% sunflower seed oil); TRT2, PS with 75 ppm NaNO_2_ and 0.1% POS.

**Table 2:** The pH and color values of boiled pork sausages with different levels of sodium nitrite and paprika oleoresin solution

Items	pH	CIE L*	CIE a*	CIE b*
Treatments^1)^				
REF	6.29±0.04^a^	73.7±0.62^b^	11.1±0.26^b^	7.79±0.41^b^
CTL	6.18±0.11^b^	75.1±0.61^a^	9.85±0.28^c^	7.77±0.25^b^
TRT1	6.29±0.06^a^	74.1±0.82^b^	12.4±0.33^a^	9.02±0.13^a^
TRT2	6.29±0.04^a^	73.6±0.21^b^	12.8±0.20^a^	8.99±0.30^a^
Storage days				
0	6.28±0.03^ab^	73.7±0.86^a^	11.5±1.17^a^	8.19±0.85^c^
7	6.31±0.01^a^	74.5±1.05^a^	11.6±1.44^a^	8.27±0.64^bc^
14	6.28±0.01^ab^	74.0±0.57^a^	11.5±1.21^a^	8.35±0.75^abc^
21	6.22±0.09^bc^	74.2±0.78^a^	11.4±1.31^a^	8.52±0.66^ab^
28	6.20±0.09^c^	74.2±0.90^a^	11.6±1.13^a^	8.63±0.55^a^

1) Treatments: REF, boiled pork sausage (BPS) with 150 ppm sodium nitrite (NaNO_2_); CTL, BPS with 37.5 ppm NaNO_2_; TRT1, PS with 37.5 ppm NaNO_2_ and 0.1% paprika oleoresin solution (POS, 5% paprika oleoresin + 95% sunflower seed oil); TRT2, PS with 75 ppm NaNO_2_ and 0.1% POS.

a–c Means (n = 3) having the same superscripts in the same column are not different (p>0.05).

**Table 3:** Purge loss (%) and TBARS (mg MDA/kg) of boiled pork sausages with different levels of sodium nitrite and paprika oleoresin solution

Items	PL	TBARS
Treatments^1)^		
REF	1.76±0.77^a^	0.15±0.66^a^
CTL	2.60±1.24^a^	0.17±0.05^a^
TRT1	1.33±0.86^a^	0.14±0.05^a^
TRT2	1.42±0.83^a^	0.14±0.05^a^
Storage days		
0	-	0.10±0.02^c^
7	1.36±0.60^a^	0.13±0.02^c^
14	1.81±0.84^a^	0.14±0.03^bc^
21	1.52±1.43^a^	0.18±0.04^ab^
28	2.42±0.93^a^	0.19±0.06^a^

TBARS, thiobarbituric acid reactive substance; MDA, malondialdehyde; PL, purge loss.

1) Treatments: REF, boiled pork sausage (BPS) with 150 ppm sodium nitrite (NaNO_2_); CTL, BPS with 37.5 ppm NaNO_2_; TRT1, PS with 37.5 ppm NaNO_2_ and 0.1% paprika oleoresin solution (POS, 5% paprika oleoresin + 95% sunflower seed oil); TRT2, PS with 75 ppm NaNO_2_ and 0.1% POS.

a–c Means (n = 3) having the same superscripts in the same column are not different (p>0.05).

**Table 4:** Residual nitrite content (ppm) in boiled pork sausages with different levels of sodium nitrite and paprika oleoresin solution

Treatments^1)^	Storage days				

	0	7	14	21	28
REF	23.9±1.99^Aa^	21.6±0.22^Aa^	13.3±1.33^Ab^	12.5±1.25^Ab^	11.4±1.10^Ab^
CTL	7.25±2.36^Ba^	4.70±0.22^Cab^	3.60±0.88^Cbc^	1.16±0.81^Bc^	0.60±0.05^Bc^
TRT1	6.27±2.58^Ba^	4.28±0.37^Cab^	2.82±1.55^Cab^	0.69±0.18^Bb^	0.45±0.03^Bb^
TRT2	12.3±0.96^Ba^	10.1±1.03^Bb^	8.40±0.74^Bb^	2.77±0.44^Bc^	2.20±0.52^Bc^

1) Treatments: REF, boiled pork sausage (BPS) with 150 ppm sodium nitrite (NaNO_2_); CTL, BPS with 37.5 ppm NaNO_2_; TRT1, PS with 37.5 ppm NaNO_2_ and 0.1% paprika oleoresin solution (POS, 5% paprika oleoresin + 95% sunflower seed oil); TRT2, PS with 75 ppm NaNO_2_ and 0.1% POS.

A–C Means (n = 3) having the same superscripts in the same column are not different (p>0.05).

a–c Means (n = 3) having the same superscripts in the same row are not different (p>0.05).

**Table 5:** Cooking loss, expressible moisture, and texture profile analysis of boiled pork sausages with different levels of sodium nitrite and paprika oleoresin solution

Items	Treatments^1)^			

	REF	CTL	TRT1	TRT2
Cooking loss (%)	1.79±0.34^a^	1.70±0.75^a^	1.81±0.45^a^	1.66±0.25^a^
Expressible moisture (%)	14.0±0.54^a^	14.7±1.08^a^	13.5±0.05^a^	14.9±0.05^a^
Hardness (gf)	4,295±510^a^	3,869±577^a^	4,614±96.6^a^	4,504±449^a^
Springiness (mm)	5.29±0.05^a^	4.84±0.23^a^	5.08±0.07^a^	4.67±0.61^a^
Gumminess	37.1±7.07^a^	40.4±10.7^a^	41.4±3.43^a^	41.0±6.17^a^
Chewiness	183±29.8^a^	147±53.3^a^	201±16.3^a^	173±19.0^a^
Cohesiveness	0.01±0.00^a^	0.01±0.00^a^	0.01±0.00^a^	0.01±0.00^a^

1) Treatments: REF, boiled pork sausage (BPS) with 150 ppm sodium nitrite (NaNO_2_); CTL, BPS with 37.5 ppm NaNO_2_; TRT1, PS with 37.5 ppm NaNO_2_ and 0.1% paprika oleoresin solution (POS, 5% paprika oleoresin + 95% sunflower seed oil); TRT2, PS with 75 ppm NaNO_2_ and 0.1% POS.

a Means (n = 3) having the same superscripts in the same row are not different (p>0.05).
